# Response of regional oxygen saturation technologies during hypoxia

**DOI:** 10.1186/cc14513

**Published:** 2015-03-16

**Authors:** UB Borg, AM Neitembach

**Affiliations:** 1 Covidien, Boulder, CO, USA

## Introduction

The purpose of this study was to determine the rate and magnitude of response to hypoxia for three different regional oxygen saturation (rSO_2_) devices. rSO_2_ technologies are focused on absolute accuracy without consideration of response characteristics. Current rSO_2_ technologies assume that the oxygen saturation is a fixed ratio of arterial and venous blood. Cerebral arteries have an oxygenation-related vasoactivity that may change the arterial/venous ratio during hypoxia. Thus, absolute rSO_2_ accuracy may be less important compared with sensitivity to changes in cerebral rSO_2_.

## Methods

Ten subjects completed the study and are included in the analysis. One INVOS sensor (SAFB-SM) was placed on the left side and one Equanox (8000CA) or Foresight (1 July 2007 or 1 July 2005) sensor (alternated between subjects) was placed on the right side of the forehead for bilateral monitoring. Desaturation was induced by adjusting the inspiratory gas mixture of O_2_/N_2_. Desaturation was titrated from room air to achieve a plateau of 70% arterial oxygen saturation (SpO_2_). Resaturation was induced by rapid change in FiO_2 _to 1.0. After 5 minutes of SpO_2_ 100%, the process was repeated by desaturation to SpO_2_ 70% and rapid return to SpO_2_ 100%. Cerebral and pulse oximetry data were recorded during the study and the time of each FiO_2_ change and plateau was recorded. rSO_2_ levels at 10, 20, 40, 60 and 80% of the total SpO_2_ response were calculated for each device to assess the rate of rSO_2_ change. The rate of rSO_2_ change in seconds and total rSO_2_ change were compared.

## Results

The rate of rSO_2_ change during desaturation was similar for all devices with an average slope factor of 0.17 for Foresight, 0.16 for Equanox and 0.21 for INVOS. The rate of rSO_2_ change in seconds during resaturation from SaO_2_ 70% to SaO_2_ 100% was significantly faster for INVOS (42 ± 16) compared with Foresight (57 ± 20) (*P *< 0.05). There was significant difference in total rSO_2_ change between INVOS (23 ± 4%) and Equanox (15 ± 4%) during desaturation and resaturation (*P *< 0.005) and between INVOS compared with Foresight (20 ± 5%) (*P *< 0.05).

## Conclusion

All rSO_2_ devices followed the SpO_2_ slope during desaturation as expected. The differences between the devices in terms of total rSO_2_ change reached statistical significance. There were also significant differences in the rate of rSO_2_ change in seconds between INVOS and Foresight during resaturation. The rate of absolute change in seconds and the magnitude of absolute change may result in better resolution to detect physiological changes. Clinical studies are required to elucidate the clinical relevance of the differences.

